# Supplementary material for: Generation of Loa loa infective larvae by experimental infection of the vector, Chrysops silacea
Source: PLoS Negl Trop Dis. 2020 Aug 17;14(8):e0008415. doi: 10.1371/journal.pntd.0008415 (PMC7470323; doi:10.1371/journal.pntd.0008415)
Supplement: Attachment — Submitted filename: PNTD-D-20-00166 R1 response to review.docx [file pntd.0008415.s001.docx]

**We thank the reviewer for taking the time to thoroughly read our manuscript and are pleased that they consider the work of interest. We have addressed the reviewers’ comments and our answers are detailed in the section below. In addition, we used tracked changes denoting where the text has been changed within the manuscript.**

**Reviewer 1**

The objectives of the study to develop a tractable model for evaluating drug efficacy is clearly articulated. The study design set forth by the authors largely addresses the objectives in testing the hypothesis. No major concerns in the results, but the absence of sham infections is missing. (see comments below).

*Results:*

Yes, the analysis is fine. Some of the figures (images) need better clarity. (see comments below)

*Summary and General Comments:*

Because the authors kind of rely on the yield/death of the flies to finally go forward with the 100 mf condition, why were sham infections not carried out?

**We did not carry out sham infections. However, given the fact that a greater proportion of *Chrysops* flies survived 50 mf compared to 100 mf injections, this indicates that mortality is related to the experimental infection with *Loa* rather than the injection procedure per se. We agree with the reviewer that we should have carried out the sham infection to empirically prove mortality is not markedly a result of our injection technique. We certainly will perform sham infections within future experiments. Nevertheless, we think that the presented results are conclusive and we have additionally discussed the missing sham infection as limitation within the discussion section (lines 474-477)**

Figure 1. Does not help. A more clear and close-up view would probably do more justice.

**We have provided an updated version of this figure.**

Figure 3. Again a better resolution will be helpful.

**We have improved the resolution of this figure.**

Figure 4g. Cannot make out the caudal papillae that the camouflaged black arrows are pointing to in the dark green background.

**We have updated of this figure.**

Ln 114. Tentative experiments - Not sure if this would apply to studies that have already been done..!!

**The sentence is now amended as follows:**

***“However, applying the latter technique for infection of Chrysops for L3 production have been unsuccessful as these flies are refractory to feeding in captivity.“ (Line 120-122)***

Methods: Lns 138-144. This is not needed in the methodology section. It is anyway mentioned again later.

**OK, we have removed the text and the associated references**

Ln 206 - Should read as 14 days post infection

**Amended**

Ln 244 - 'eutheised by rising CO2': should it be euthanized with CO2?

**Amended to *“euthanised by a rising concentration of CO2”***

**Reviewer 2**

The objectives of the study are clearly articulated, the study design is appropriate. The sample size if acceptable. Statistical analysis requires minor revision.

I understand that the ethics and protocol were approved and received clearances from the a committee devoted to the Institution where the work was conducted (REFOTDE). I don't know if it was done this way because there is no alternative in the country or for other reasons. Some clarifications (in the cover letter at least) would be informative to readers unfamiliar with the regulation in the country where it was done (Republic of Cameroon).

**The REFOTDE INSTITUTIONAL review board (RIAEC) was created in 2005, with the assistance of consultant from the Veterinary School of the Michigan State University (MSU). This institutional review board, reviews protocols from researchers from different academic institutions in Cameroon. They board is independent and avoid conflict of interest in their activities. RIAEC has been amongst the first institutional review board that review research protocols involving animal use for biomedical research in Cameroon.**

*Results:*

Table 2: the first four columns are identical to those of table 1. I suggest merging the two tables to avoid redundancy.

**Amended according to reviewer’s correction.**

It would be interesting to know, for each group, the percentage of chrysops with L3s.

**For all *Chrysops* dissected, we obtained at least ≥ 2 L3 recovered for each fly (100%). We have indicated this in the text now (Table 2 and lines 330-331).**

Line 332: I can't figure out where the denominator (2453) comes from. Please clarify.

**We did not use all larvae and cultured only 2453. We have just included a sentence about this information in lines 394-396**

***“Of the 2462 L3 recovered from flies that were injected with 50 mf, 2453 were successfully placed into culture. Subsequently, 1450/2453 larvae underwent moulting (from L3 stage to L4 stage; Fig.3 & Table 3).”***

Line 335: Cuzick test for trend is more appropriate than a Chi2 test.

**Amended according to reviewer’s correction (line 398). We have also updated the section on data analysis accordingly (lines 301-306).**

Lines 347-351: this should be in the method section.

**Amended**

In table 5: how many L3 were injected per mice? This should be given as supplementary material.

**Amended according to reviewer’s correction**. **A table is provided as supplements indicating the number of larvae injected to each animal.**

*Conclusions:*

The conclusion is supported by the data presented. Some limitations are addressed. A section addressing the cost could be interesting.

**We appreciate the reviewer’s thought. A detailed cost analysis has not been performed but we predict costs of experimental procedures will be offset by reduced human hours required for fly dissections.**

*Editorial and Data Presentation Modifications:*

Line 250: add "." after giving the 2 refs.

Line 92: I suggest adding "during" between "post-ivermectin" and "mass treatment".

Lines 95-99: the sentence is really (too) long, the authors should split it. In addition, the new WHO roadmap now targets onchocerciasis elimination by 2030.

Line 145: "were" seems missing before "undertaken".

**Amended according to reviewer’s correction.**

Line 255: ref 28 is cited before refs 26 and 27.

**The citation and bibliography have been updated.**

Discussion:
Lines 382 - 391: this part is redundant with the introduction. It is generic. The authors should go up to the point of their own work.

**The redundancy is removed.**
